# Diagnosis and treatment of penile verrucous carcinoma

**DOI:** 10.3892/ol.2015.2909

**Published:** 2015-01-27

**Authors:** FANGYIN LI, YIPENG XU, HUA WANG, BO CHEN, ZONGPING WANG, YANG ZHAO, SHAOXING ZHU, GUIPING CHEN

**Affiliations:** 1 Department of Urology, Zhejiang Cancer Hospital, Hangzhou, Zhejiang 310022, P.R. China; 2 Department of Pathology, Zhejiang Cancer Hospital, Hangzhou, Zhejiang 310022, P.R. China

**Keywords:** penile verrucous carcinoma, condyloma acuminatum, surgical treatment, diagnosis

## Abstract

Penile verrucous carcinoma is an extremely rare disease that, at present, has not been well characterized. The etiology, diagnosis and treatment of this carcinoma remain poorly understood, particularly in the Chinese population. The aim of the present study was to discuss the methods of diagnosis and treatment of penile verrucous carcinoma in the Chinese population. The clinical and pathological data of 10 patients with penile verrucous carcinoma were analyzed alongside a literature review. All the tumors were exophytic papillary lesions, ranging between 0.4 and 4 cm in diameter and all 10 patients underwent partial penectomy with tumor-negative surgical margins. None of the 10 patients underwent ilioinguinal lymphadenectomy. All patients were regularly followed up for 0.7–9 years, which revealed that no patients developed recurrence, and only one case resulted in mortality due to unassociated causes. It was found that penile verrucous carcinoma is a well-differentiated disease with low malignant potential and locally aggressive features, which seldom metastasizes to regional lymph nodes or distant regions. However, misdiagnosis may occur due to an incorrect biopsy. Favorable outcomes can be achieved by surgery, even without any adjuvant therapy, but patients should be carefully followed up.

## Introduction

The incidence of penile carcinoma varies between countries, with the highest morbidity in Africa and lowest in the USA and Europe (1). The causes of this variation remain unclear, but appear to be associated with epidemiological factors and public health conditions. According to a previous study, the morbidity associated with penile carcinoma is correlated with poor education, a low income, inhabitance of a rural region, a history of heavy smoking, chronic inflammation, genital warts, penile tears, frequent phimosis and poor genital hygiene habits (1). Penile carcinoma has been the most common male urogenital malignant tumor in China since the 1950s (2). Concurrent with the economic development and the improvement of sanitary conditions, the incidence of penile carcinoma has reduced gradually in China, with a current morbidity rate similar to that of western countries. Penile carcinoma accounts for ≤0.5% of all male carcinoma cases in Europe and the USA, the majority of which are squamous cell carcinomas (SCCs) (3). The most common type of SCC of the penis is conventional SCC, accounting for 48–65% of cases, followed by basaloid carcinoma, accounting for 4–10% of cases, warty carcinoma, which accounts for 7–10% of cases and verrucous carcinoma, which accounts for 3–8% of cases (1).

Penile verrucous carcinoma, a variant of well-differentiated SCC, is characterized by slow growth and a locally aggressive nature, but it rarely metastasizes to regional nodes or distant regions (4,5). This carcinoma is an extremely rare disease and is not well characterized (6). The etiology, diagnosis and treatment of penile verrucous carcinoma remain poorly understood, particularly in the Chinese population. At present, the mainstay for treatment of penile verrucous carcinoma continues to be penectomy, which generally leads to psychosexual issues and markedly diminishes the quality of life. Other therapies include intra-aortic infusion chemotherapy, which has been demonstrated to be effective and is considered as an organ-sparing treatment, particularly for younger patients (4). In the present study, the tissues obtained by penectomy from 10 cases of penile verrucous carcinoma were retrospectively analyzed to assess the methods of diagnosis and treatment of penile verrucous carcinoma in the Chinese population.

## Patients and methods

### Patients

In total, 10 cases of penile verrucous carcinoma treated at the Zhejiang Cancer Hospital (Hangzhou, Zhejiang, China) were retrospectively analyzed. The patients were diagnosed and treated in the hospital between December 1999 and December 2009. The age of the patients ranged between 35 and 72 years old, with a mean age of 51.5 years. Eight of the patients noticed a penile mass and the other two were found to possess a neoplasm on the glans penis when they underwent circumcision. All patients presented with redundant prepuce or phimosis. The tumor diameter ranged between 0.8 and 4 cm. All tumors exhibited exophytic growth that appeared cauliflower-like. The lesions involved the entoplastron of the prepuce in six patients and the glans in four patients. Only one patient presented with ulcers and local pain due to infection. Neither inguinal lymph node nor distant metastasis was observed using chest radiography or ultrasonic examination. All patients provided written informed consent.

### Methods

Diagnosis of penile verrucous carcinoma mainly relies on biopsy. In the present study, eight patients underwent a biopsy examination to obtain a definite diagnosis prior to surgery. One of these patients received a biopsy examination due to phimosis, but only a small sample of protruding tissue at the osculum of the prepuce was removed and was pathologically diagnosed as papilloma. An additional surgical exploration was subsequently performed on this patient and a tumor with a diameter of ~4 cm involving the entoplastron of the prepuce was found. Circumcision was performed again. The histological examination revealed the lesion to be penile verrucous carcinoma and partial penectomy was subsequently performed.

One of the remaining two patients underwent circumcision at Cangan People’s Hospital (Wenzhou, China) due to a mass in the entoplastron of the prepuce. The histological examination revealed the lesion to be verrucous carcinoma. Three months later, a nodule was found in the coronary sulcus of the penis. Biopsy examination at the Zhejiang Cancer Hospital also revealed the tumor to be penile verrucous carcinoma and the patient then underwent a partial penectomy.

The remaining patient had undergone a circumcision due to phimosis in 1999. During the surgery, a cauliflower-like neoplasm was found at the dorsal of glans nearing the coronary sulcus, which was hypothesized to be condyloma acuminatum and was excised locally. However, the histological examination revealed the tumor to be squamous metaplasia with partial hyperkeratosis. The papillomatous tumor recurred and was removed by partial penectomy in 2002 and in 2004, respectively. The biopsy specimens yielded similar findings. In 2006, the papillomatous tumor recurred again and the histological examination revealed the presence of verrucous carcinoma. Partial penectomy was performed.

All 10 patients received surgical treatment, eight of which received partial penectomy. This included one patient who underwent a partial penectomy due to penile verrucous carcinoma three months after undergoing circumcision for phimosis, and one patient who underwent several local excisions, as aforementioned, and then underwent a partial penectomy. No patients received ilioinguinal lymphadenectomy or chemotherapy.

## Results

**Figure 1:**
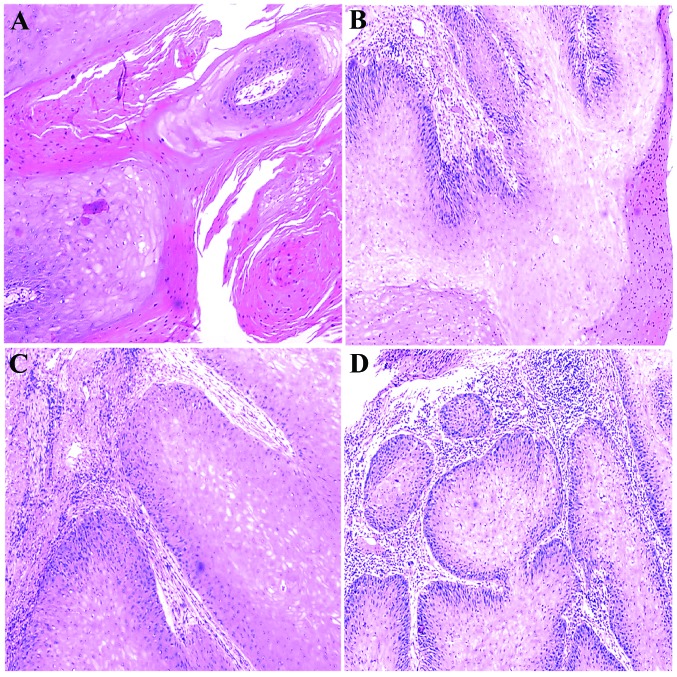
The microscopic examination of penile verrucous carcinoma tissue obtained from different patients. (A) Tumor cells growing in the corpora mammillaria demonstrating hyperkeratosis and parakeratosis. (B) Tumor cells growing in the corpora mammillaria demonstrating acanthosis and less atypia. (C) Tumor cells extending downwards in a regular manner. The parenchyma was drumstick-like with a clear boundary and rare distinct invasiveness was observed. (D) The base of the tumor was bulbous-like, with mild atypia cells and rich lymphocytic infiltration in the surrounding mesenchymal cells. Stain, hematoxylin and eosin; original magnification, ×50.

The surgical specimens were histologically examined at the Department of Pathology (Zhejiang Cancer Hospital). All the tumors revealed exophytic papillary lesions with a brittle texture and the incisal surface was gray. Microscopic examination revealed the tumor to be characterized by papillary growth at the surface and locally aggressive invasion in the basement membrane of the tumor. The tumor cells were well-differentiated and exhibited little heteromorphism, with rare karyokinesis. The epithelium presented rod-like interdigitation, hyperkeratosis and a papilloma-like structure (Fig. 1A and B). The epithelium grew downward into the underlying tissues in a bulbous or drumstick process. Generally, the tumor exhibited clear boundaries and rich lymphocytic infiltration in the surrounding mesenchyme (Fig. 1C and D). The patients were followed up for 8 months to 9 years. With the exception of one patient that succumbed unassociated causes, the patients presented no tumor recurrence or metastasis at the end of the follow-up.

## Discussion

Verrucous carcinoma, which was first described in 1948, has been reported in the oral cavity, anus, penis and female genitalia (7–10). This carcinoma is an extremely rare low-grade SCC that exhibits slow invasive growth and a lack of metastasis, with penile verrucous carcinoma being the most common type (11).

Penile verrucous carcinoma is a rare disease, accounting for 2.4–24% of penile cancer (12,13), which can occur in any part of the penis, but mainly occurs in the glans penis. Phimosis and redundant prepuce are two important causative factors for penile verrucous cancer (14,15). The 10 patients enrolled in the present study all suffered from phimosis or redundant prepuce, with the most common clinical manifestation being a local exogenous mass. It is challenging to identify penile verrucous cancer due to the exterior features appearing extremely similar to those of condyloma acuminatum. Penile verrucous carcinoma lesions often present as cauliflower- or wart-like, and do not cause significant pain. However, verrucous carcinoma grows slowly, without inhibition, and regions of the carcinoma can invade the glans or even the shaft. Certain larger penile verrucous carcinoma tumors possess an unpleasant odor and result in pain due to necrosis and infection. The penile verrucous carcinoma tumor cells are well-differentiated and are often accompanied by squamous epithelial hyperplasia and keratinization. Thus, verrucous carcinoma may be easily misdiagnosed if an inappropriate biopsy were to be performed. One patient in the present study was misdiagnosed with penile papilloma due to the tissue being inappropriately extracted at the distal end of the osculum of the prepuce. Therefore, deeper biopsies are recommended, according to the tumor size, and the basement membrane of the papillomatous tumor should be particularly considered during the sampling. For patients that are highly likely to possess verrucous carcinoma, but have not been diagnosed with the carcinoma, repeated biopsies should be undertaken. Occasionally, the prepuce may be opened to obtain suitable tissues if the lesion is accompanied by phimosis. However, obtaining a diagnosis by histological examination remains to be challenging if the patient suffers from giant condyloma acuminatum (11).

Previous studies have indicated that penile carcinoma is not only associated with human papilloma virus (HPV) infection, but is also correlated with other factors, including phimosis, chronic inflammation and lichen sclerosus (12,13). HPV is considered to be closely associated with penile cancer and condyloma acuminatum, which is involved in almost all cases of penile verrucous cancer (16). However, various types of HPV have been identified. Detection based on polymerase chain reaction technology identified that the type of HPV involved in penile verrucous cancer is a high-risk virus with high carcinogenicity, while the type of HPV is usually low-risk in condyloma acuminatum. Thus, for giant condyloma acuminatum that is challenging to identify, identification of the HPV type may aid in diagnosis (11). Studies have found that local squamous epithelial hyperplasia and hornification may be important for the development of penile verrucous carcinoma. In the present study, nodules recurred four times on the glans penis in one patient, and the first three histological examinations identified the tumor as squamous epithelial hyperplasia and hyperkeratosis, which was consistent with the literature (12,13).

The literature on verrucous carcinoma mostly focuses on case reports and rarely on large-scale studies. Nevertheless, surgical treatment for penile verrucous carcinoma has been generally accepted as the mainstay for treatment. Provided that the penile verrucous carcinoma is well-differentiated and exhibits good biological behavior, maximized retention of the appearance and function of the penis is an accepted surgical principle. Therefore, a wide range of local and partial resections of the penis are the most common surgical approaches, and full penectomy is seldom used in the clinic. However, due to the relatively rare incidence of penile verrucous carcinoma, surgeons often lack experience, which leads to the unnecessary removal of part or the entirety of the penis. If the margin is tumor-positive, the resection should be extended. Penile verrucous carcinoma exhibits a potential for recurrence, but the incidence rates vary between studies. If the carcinoma recurs repeatedly, the patient may require an additional resection or even full penectomy.

Shimizu *et al* (17) found that ~30% of verrucous carcinomas are complicated with micro-lesions of invasive squamous cell carcinoma, and that certain lesions will eventually progress to other types of invasive squamous cell carcinoma. A previous study (18) proposed that urethral lesions may be an early event in the carcinogenesis of penile cancer, as they appear on the head of the penis at the early stage of penile cancer. Patients undergoing local excision should pay additional attention to the recurrence of the lesion and be followed-up closely. If any sign of recurrence is observed, the penis should be further partially resected or totally removed. As almost no distant metastasis is found in patients with verrucous carcinoma, inguinal lymphadenectomy is seldom performed.

Previously, inguinal lymphadenectomy was performed on certain patients, however, no evident lesions were found (19). Thus, inguinal lymphadenectomy is not recommended as a prophylactic treatment. As for patients with localized inguinal lymphadenectasis, anti-infection treatment may be undertaken initially and, if necessary, lymph node biopsy may be performed. In the present study, all 10 patients underwent surgical treatment with the retention of the penis, but did not undergo lymph node dissection. With the exception of one patient who succumbed to unassociated causes, the patients had not experienced recurrence or metastasis at the end of the follow-up. This study highlighted the clinical and pathological features of penile verrucous carcinoma and its treatment. It was found that partial penectomy may result in a good prognosis and outcome. The study has provided a basis for further investigation regarding the diagnosis and treatment of penile verrucous carcinoma.
